# The Book World of Medicine and Science

**Published:** 1903-12-26

**Authors:** 


### The Book World of Medicine and Science.

The Practice of Obstetrics. Designed for the Use of Students and Practitioners of Medicine. By J. Clifton Edgar, Professor of Obstetrics in the Cornell University Medical College (London: Rebman, Limited. 1,111 pages and 1,221 illustrations. Price £1 10s. net)

Of recent years a number of valuable text-books have been published by American obstetricians; these have been distinguished by the number and beauty of their illustrations, and by the patient industry and wide reading displayed by their authors, but they for the most part exhibit a tendency to develop the more purely scientific aspects of the questions with which they deal at the expense of the practical side. These characteristics mark the book before us. Professor Edgar has written a volume for which all who are engaged in the teaching of obstetrics will be very grateful to him, but it is as a book of reference rather than as a text-book for students that it commands our admiration. For the average student the mass of detail here given will prove bewildering, and the broad principles which should guide him in his treatment are not sufficiently emphasised. Take for instance the section upon accidental hæmorrhage, one page only is devoted to the treatment, and that page is practically a catalogue of different methods, amongst which the reader is left to take his choice, and furthermore is soothed into a feeling of false security by such comforting sentences as "the most efficient check to hæmorrhage is uterine contraction which must be brought about if possible. By rupture of the membranes the liquor amnii will escape, and the uterine contraction will take place." Few are the cases of severe accidental hæmorrhage in which this happy result is obtained by rupture of the membranes. The arrangement of the book differs somewhat from that usually followed by authors of text-books. The customary chapter on anatomy is omitted, but ovulation, menstruation, and other matters not usually discussed in works upon obstetrics are considered here. The sections on pregnancy have been carefully written, and many original pictures of value are given. Some, however, are unnecessary, and might well be dispensed with, such as the whole-page illustration of the anti-partum bath. Deciduoma malignum is still classed amongst diseases of the decidua, although the author apparently accepts its fœtal origin. The account of its histology is very inadequate. "Its histological structure resembles in general that of placental tissue pursuing an atypical course, and bears the same relation to normal placenta as a carcinoma of the breast bears to the normal mammary gland" is all that is said on this very important matter. The illustration of its structure given on page 207 is absolutely valueless. Toxæmia of pregnancy is discussed under the heading of diseases of the urinary tract, and in one chapter we find grouped together such different conditions as chronic nephritis and eclampsia. This arrangement is unfortunate, the old belief in the renal origin of eclampsia dies hard and it is necessary still to make it very clear to the mind of the student that uræmia and eclampsia are totally different conditions. Upon this chapter a great amount of care has evidently been bestowed, but there is a lack of proportion and a lack of clearness; nephritis commands only two short paragraphs, the sections upon "The Kidney of Pregnancy" and "Renal Insufficiency" are vague and very speculative, whilst that on eclampsia itself makes no mention of the later experimental work or certain more recent modes of treatment. A section of 50 pages on anti-natal pathology is a new feature in a text-book; the author tells us that for the most part it was written before Dr. Ballantyne's excellent book appeared, and it forms therefore one of the first systematic accounts of this important branch of study. It contains a mass of facts, is well written and profusely illustrated. Labour and the puerperium receive their due amount of attention, and the chapter devoted to obstetric operations is one of particular merit. We can congratulate Dr. Edgar upon having written a really valuable book. To the English student it will not form a safe examination guide, for in many essential matters English and American practice differ widely, but as a book of reference, and as a picture of the present condition of obstetrics in America, it fills an important place. The execution of the drawings cannot be too highly praised, and although some of the pictures appear to be superfluous, the majority of them are of real value. Dr. Edgar is to be congratulated also upon his publisher; the printing and arrangement of the book leave nothing to be desired.

Digest of Researches and Criticisms Bearing on the Revision of the British Pharmacopœia, 1898. By W. Chattaway, F.I.C. (London: 1903).

This is a valuable compendium of various criticisms, researches, and suggestions published from the beginning of the year 1899 to the end of 1902, which would be useful for the revision of the British Pharmacopœia of 1898. The work, which was carried out under the direction of the pharmacopœia committee of the General Council of Medical Education and Registration of the United Kingdom, has entailed a great labour on the compiler, and Mr. Chattaway is to be congratulated on the clearness and brevity of the abstracts which he has made. He notes that the researches deal mainly with the composition and assay of drugs and galenicals, while criticisms dealing with the botanical features of drugs are remarkably few. The book should prove valuable to pharmacologists and others for purposes of reference.

Year-Book of the Scientific and Learned Societies of Great Britain and Ireland. Compiled from official sources. Twentieth Annual Issue. (London: Charles Griffin & Co. 1903. Price 7s. 6d.)

This year-book should prove very useful to those interested in the evolution of modern science. It contains a list of the various learned societies with their addresses, the names of their officers, and their places of meeting, together with the titles of the papers recently read before them or published under their auspices.

Doctor's Diary and Emergency Note-book, 1904. (London: Scott and Bowne, Ltd.)

This is a very handy little note-book. It is small enough to fit into the waistcoat pocket, and yet contains sufficient space for registering appointments and making memoranda, besides giving much information which might prove useful to the busy practitioner; for example, the addresses are given of the various consumption sanatoria, lunatic and idiot asylums, and inebriate homes in the British Isles, besides valuable hints on other matters such as certificates and coroners, wills, infectious diseases, and so forth.

